# TRPV2: A Key Player in Myelination Disorders of the Central Nervous System

**DOI:** 10.3390/ijms23073617

**Published:** 2022-03-25

**Authors:** Jennifer Enrich-Bengoa, Gemma Manich, Tony Valente, Paula Sanchez-Molina, Beatriz Almolda, Carme Solà, Josep Saura, Berta González, Bernardo Castellano, Alex Perálvarez-Marín

**Affiliations:** 1 Biophysics Unit, Department of Biochemistry and Molecular Biology, School of Medicine, Universitat Autònoma de Barcelona, 08193 Cerdanyola del Vallès, Catalonia, Spain; jennifer.enrich@uab.cat; 2 Institut de Neurociències, Universitat Autònoma de Barcelona, 08193 Cerdanyola del Vallès, Catalonia, Spain; gemma.manich@uab.cat (G.M.); tony.valente@uab.cat (T.V.); paula.sanchez@uab.cat (P.S.-M.); beatriz.almolda@uab.cat (B.A.); berta.gonzalez@uab.cat (B.G.); bernardo.castellano@uab.cat (B.C.); 3 Medical Histology Unit, Department of Cell Biology, Physiology and Immunology, School of Medicine, Universitat Autònoma de Barcelona, 08193 Cerdanyola del Vallès, Catalonia, Spain; 4 Research Group on Methodology, Methods, Models and Outcomes of Health and Social Sciences (M3O), Experimental Sciences and Methodological Department, Faculty of Health Sciences and Welfare, University of Vic-Central University of Catalonia (UVic-UCC), 08500 Vic, Catalonia, Spain; 5 Department of Cerebral Ischemia and Neurodegeneration, Institut D’Investigacions Biomèdiques de Barcelona-Consejo Superior de Investigaciones Científicas (CSIC), Institut D’Investigacions Biomèdiques August-Pi i Sunyer (IDIBAPS), 08036 Barcelona, Catalonia, Spain; carme.sola@iibb.csic.es; 6 Biochemistry and Molecular Biology Unit, School of Medicine, Institut D’Investigacions Biomèdiques August-Pi i Sunyer (IDIBAPS), University of Barcelona, 08036 Barcelona, Catalonia, Spain; josepsaura@ub.edu

**Keywords:** transient potential receptor vanilloid 2, Opalin, oxidative stress, myelination, multiple sclerosis, recapitulation theory

## Abstract

Transient potential receptor vanilloid 2 (TRPV2) is widely expressed through the nervous system and specifically found in neuronal subpopulations and some glial cells. TRPV2 is known to be sensitized by methionine oxidation, which results from inflammation. Here we aim to characterize the expression and regulation of TRPV2 in myelination pathologies, such as hypomyelination and demyelination. We validated the interaction between TRPV2 and its putative interactor Opalin, an oligodendrocyte marker, in mixed glial cultures under pro- and anti-inflammatory conditions. Then, we characterized TRPV2 time-course expression in experimental animal models of hypomyelination (jimpy mice) and de-/remyelination (cuprizone intoxication and experimental autoimmune encephalomyelitis (EAE)). TRPV2 showed upregulation associated with remyelination, inflammation in cuprizone and EAE models, and downregulation in hypomyelinated jimpy mice. TRPV2 expression was altered in human samples of multiple sclerosis (MS) patients. Additionally, we analyzed the expression of methionine sulfoxide reductase A (MSRA), an enzyme that reduces oxidated methionines in TRPV2, which we found increased in inflammatory conditions. These results suggest that TRPV2 may be a key player in myelination in accordance with the recapitulation hypothesis, and that it may become an interesting clinical target in the treatment of demyelination disorders.

## 1. Introduction

Transient potential receptor vanilloid 2 (TRPV2) is a thermo-, mechano- and lipid sensor non-selective cation channel, and is a member of the superfamily of TRP channels [1]. Activation of TRPV2 is triggered by noxious heat (>52 °C), but also by mechanical stimuli, different chemical compounds such as probenecid [1], and by oxidative stress through methionine (Met) oxidation [2]. TRPV2 is ubiquitously expressed in many human tissues, such as skeletal muscle, heart, lung, skin, thyroid, testis, and immune tissues [1,3]. In the central nervous system (CNS), TRPV2 expression has been specifically found in medium-to-large neurons bearing myelinated fibers [4], in microglia [5], astrocytes [6] and, at the mRNA level, in oligodendrocyte precursor cells (OPCs) [7], but so far, not in oligodendrocytes. TRPV2 expression and activity, both at the plasma membrane and/or internal endosomes, has been reported to be necessary for axon and neurite outgrowth of motor and sensory neurons [8,9], and in microglia and macrophages has been consistently related to phagocytosis of cellular debris [10]. 

Previous work from our lab [11] showed that TRPV2 putatively interacts with important key myelin proteins (i.e., proteolipid protein 1 (PLP1), Opalin, neurotrimin (NTM) and with other proteins involved in neoplasms and CNS diseases (ABR, FGF1, KCNJ10, PEBP1, PLP1 and SCD3) [12]. Intriguingly, Opalin, a protein located in the paranodal loop and soma of oligodendrocytes [13], was one of the proteins that showed the strongest interaction with TRPV2, suggesting that TRPV2 could be related to myelination, although its expression has not been shown in oligodendrocytes [7]. Studies by Hainz et al. [14,15,16] demonstrated that probenecid, a pannexin-1 antagonist, but also a specific TRPV2 agonist [1], improves the outcome in two experimental mouse models of multiple sclerosis (MS): in the experimental autoimmune encephalomyelitis (EAE) model, it is capable of preventing and arresting the progression of clinical symptoms and promoting oligodendrocyte proliferation [14,15] and in the cuprizone model of demyelination/remyelination, it reduces demyelination [16]. This evidence points to a putative role of TRPV2 as an upstream key player in myelination and remyelination processes in the CNS. 

In this study, we characterized the TRPV2–Opalin interaction in vitro and in mixed glial cell culture to assess if TRPV2 is expressed in oligodendrocytes. TRPV2 presence in oligodendrocyte-lineage cells, in addition to neurons, astrocytes and microglia, may provide support for the importance of TRPV2 in neuron-glial cells’ [7] cross-talk in CNS myelination. In parallel, we characterized TRPV2 expression in the CNS in three different myelination murine models: the hypomyelinating jimpy mice as a pathophysiological model for Pelizaeus–Merzbacher disease (PMD) [17,18,19] and the cuprizone- and EAE-induced experimental mouse models. To assess the potential role of TRPV2 in human pathophysiology, we determined TRPV2 gene and protein expression in tissue samples of human MS patients. 

## 2. Results

### 2.1. Opalin Interacts In Vitro with the Ankyrin-Repeat Domain (ARD) of TRPV2

**Figure 1:**
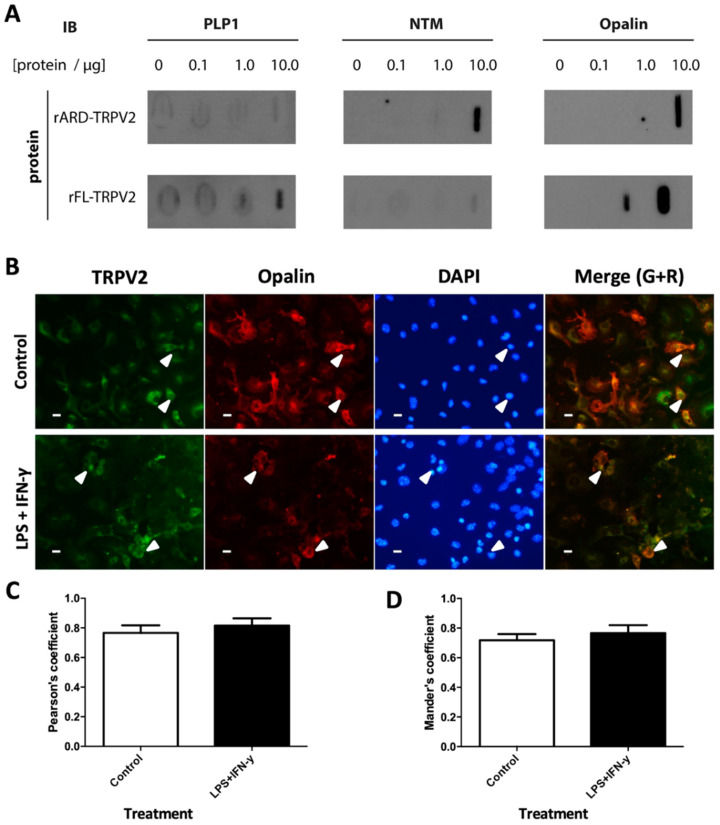
TRPV2 interaction with Opalin, NTM and PLP1 in vitro during basal and inflammatory conditions. (**A**) Rat TRPV2 FL protein and rat ARD of TRPV2 were immobilized on membranes and incubated with increasing concentrations of rat protein brain lysates (0–10 µg). Opalin, NTM and PLP1 were detected by immunoblot. (**B**) Immunocytochemistry in mouse mixed glial primary cultures shows TRPV2 (green), Opalin (red) and DAPI (blue). TRPV2-Opalin colocalization is shown as yellow signal (merge) and both proteins are co-expressed in some cells (arrowheads). Expression of these proteins is mainly found in the membrane. (**C**) TRPV2–Opalin colocalization expressed as Pearson’s coefficient in basal and inflammatory conditions. Both proteins show a high degree of colocalization. (**D**) TRPV2 colocalization with Opalin, expressed as Mander’s coefficient M2 (fraction of TRPV2 signal overlapping Opalin) in basal and inflammatory conditions. Both proteins show a high degree of colocalization. Results were expressed as mean ± SEM. Statistical analysis of the results were performed with an unpaired Student’s *t*-test (*p* < 0.05 is statistically significant). Scale bar = 20 µm.

Since previous results from our group indicated a possible interaction between TRPV2 and NTM, PLP1 and Opalin [11], we studied these interaction pairs in vitro. Purified full-length (FL) rat TRPV2 and rat ARD were immobilized on nitrocellulose membranes, and then rat brain protein extracts were incubated with the membranes. After immunoblotting against the selected interactors, we observed that Opalin interacts with both the whole protein and with the cytosolic ARD of TRPV2 in a stronger fashion than NTM and PLP1 protein (Figure 1A). The mode of interaction between FL and the cytosolic domain of TRPV2 and Opalin indicates intracellular/transmembrane interaction, so both proteins coexist in the same cell, such as an oligodendrocyte-like cell.

### 2.2. TRPV2 and Opalin Display a High Degree of Colocalization in Mixed Glial Cultures Enriched in Oligodendrocytes

Next, to assess the possibility that TRPV2 and Opalin co-express in the same cell type, we determined if they were expressed in vitro in mixed glial cell cultures containing microglia, astrocytes and oligodendrocytes (Supplementary Figure S1). Co-expression was determined in basal conditions and after a pro-inflammatory stimulus, since myelination disorders are associated with inflammatory processes. Results showed that some TRPV2+ cells also expressed Opalin with a high degree of colocalization (Figure 1B), as indicated by ca. 0.8 Mander’s and Pearson’s coefficients (Figure 1C,D). After exposure to a pro-inflammatory stimulus such as lipopolysaccharide (LPS) + interferon-γ (IFN-γ), levels of colocalization remained unchanged (Figure 1C,D).

### 2.3. TRPV2 Expression in Microglia and Oligodendrocytes Is Regulated after Pro-Inflammatory, Anti-Inflammatory and Demyelinating Treatments

**Figure 2:**
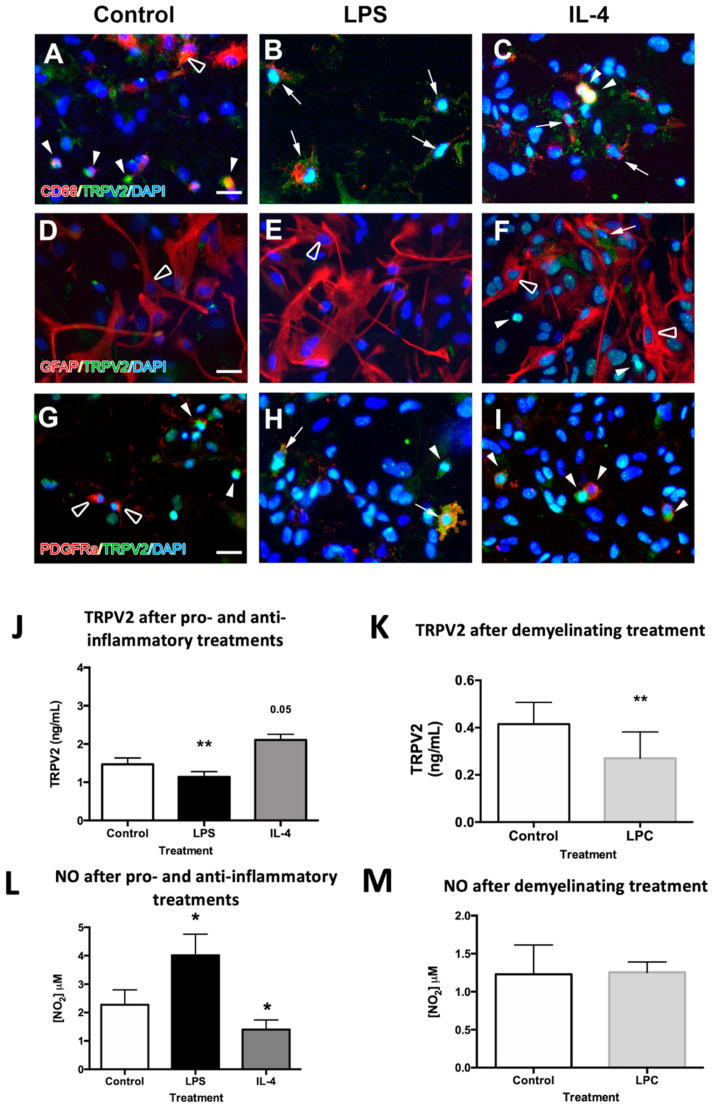
TRPV2 expression in mixed glia cultures of mice after pro-inflammatory (LPS), anti-inflammatory (IL-4) and demyelinating (LPC) treatments. (**A**–**I**) Double immunohistochemical staining allowed for the determination of whether TRPV2 was expressed in microglia (**A**–**C**), astrocytes (**D**–**F**) or oligodendrocyte cells (**G**–**I**). TRPV2 was highly expressed in the cell body of some microglia (full arrowheads) but not all of them (empty arrowheads) in control conditions, and TRPV2 was spread afterwards through the cell body of activated microglia cells, with an expression pattern highly coincident with CD68+ lysosomes (arrows). While no expression of TRPV2 was found in astrocytes (empty arrowheads), OPCs showed either low or absent expression in control conditions (full and empty arrowheads, respectively) and increased expression throughout the cell body (arrows) and the principal soma (arrowheads) after both pro-inflammatory and anti-inflammatory treatments. (**J**,**K**) Quantification of TRPV2 protein (ng/mL) by ELISA in secondary mixed glial cultures in control conditions, and after LPS and IL-4 treatments (**J**) and in quaternary mixed glial cultures, containing myelin-binding protein (MBP)+ oligodendrocytes, in demyelinating conditions (**K**). Determination of TRPV2 showed a significant decrease in TRPV2 after LPS and LPC treatments (**J**,**K**), and an increase after IL-4 treatment (*p* = 0.05) compared to control conditions (**J**). (**L**,**M**) Quantification of NO secondary mixed glial cultures in control conditions, and after LPS and IL-4 treatments (**L**) and in quaternary mixed glial cultures in demyelinating conditions (**M**). An increase in NO concentration was observed in cell cultures after LPS treatment compared to control conditions, while a significant decrease was observed after IL-4 treatment. After LPC treatment, NO remained stable compared to the control. Statistical analysis was performed by one-way ANOVA followed by Dunnett’s multiple comparison compared to the control in the LPS and IL-4 treated cultures (* *p* < 0.05, ** *p* < 0.01), and a paired Student’s *t*-test for LPC treatment (** *p* < 0.01). Scale bar (**A**–**I**) = 25 µm.

Cellular expression of TRPV2 was determined with double immunocytochemical staining performed in glial mixed cultures. These cultures were composedof approximately 25% microglia, 30% astrocytes and 35% oligodendrocytes in basal conditions, and their populations varied slightly after pro-inflammatory treatment (Supplementary Figure S1). In basal conditions, TRPV2 was mainly expressed in microglia, detected by lysosomal CD68, and oligodendrocytes, identified by platelet-derived growth factor receptor alpha (PDGFRα) (Figure 2A,G), while no expression was observed in glial fibrillary acidic protein (GFAP)-labelled astrocytes (Figure 2D). In both microglia and oligodendrocytes, TRPV2 was highly expressed and concentrated in the cell body, without labelling in their ramifications. After the pro-inflammatory treatment with LPS, TRPV2 intensity decreased in activated microglia, but its expression was extended to all of the microglial cell body and membrane, with a punctate staining similar to lysosomal CD68 (Figure 2B). In oligodendrocytes, we observed a similar expression pattern, consisting of a decreased intensity of TRPV2 staining spread throughout PDGFRα+ cell bodies (Figure 2H). After interleukin (IL)-4 anti-inflammatory treatment, TRPV2 was found in both ramified and activated microglia phenotypes, following the expression patterns explained above (Figure 2C). Similarly, oligodendrocytes showed high TRPV2 expression in their cell bodies, as been observed in the control condition (Figure 2I). In none of the treatments did GFAP+ astrocytes show TRPV2 expression (Figure 2E,F). 

Quantification of total TRPV2 expression in mixed glial cultures showed a significant decrease of TRPV2 after LPS pro-inflammatory treatment (Figure 2J, *p* < 0.01) compared to controls. After an anti-inflammatory IL-4 treatment, TRPV2 expression tended to increase (Figure 2J, *p* = 0.05) when compared to controls. Oxidative stress is known to induce oxidation of specific Met residues in TRPV2 (Met528 and Met607 in rat TRPV2) [2,20], endogenously activating the channel. As an indicator of oxidative stress in the cell culture, we measured nitric oxide (NO) concentration in cell media. Indeed, NO is also interesting since it promotes TRPV2 translocation to the plasma membrane [5]. Results showed that IL-4 treated cultures presented lower levels of NO in the cell culture medium compared to the control, while LPS-treated cultures showed a significant increase of NO (Figure 2L).

In an attempt to closely determine the TRPV2 response in demyelination processes, we treated quaternary glial mixed cell cultures containing myelin-binding protein (MBP)-positive mature oligodendrocytes (see cell populations in Supplementary Figure S2) with the demyelinating and apoptotic cell agent L-α-lysophosphatidylcholine (LPC), which is also a direct activator of TRPV2 [21]. TRPV2 levels were significantly decreased in LPC-treated cultures compared to their respective controls, as similarly observed in the LPS pro-inflammatory treatment (Figure 2K, *p* < 0.01). Determination of NO in the cell media of LPC-treated cells, as a measure of oxidative stress, demonstrated similar levels compared to the control condition (Figure 2M).

### 2.4. TRPV2 and Opalin Expression, but Not Methionine Sulfoxide Reductase A (MSRA) Levels, Are Importantly Reduced in the Hypomyelinating Jimpy Mutant Mice

**Figure 3:**
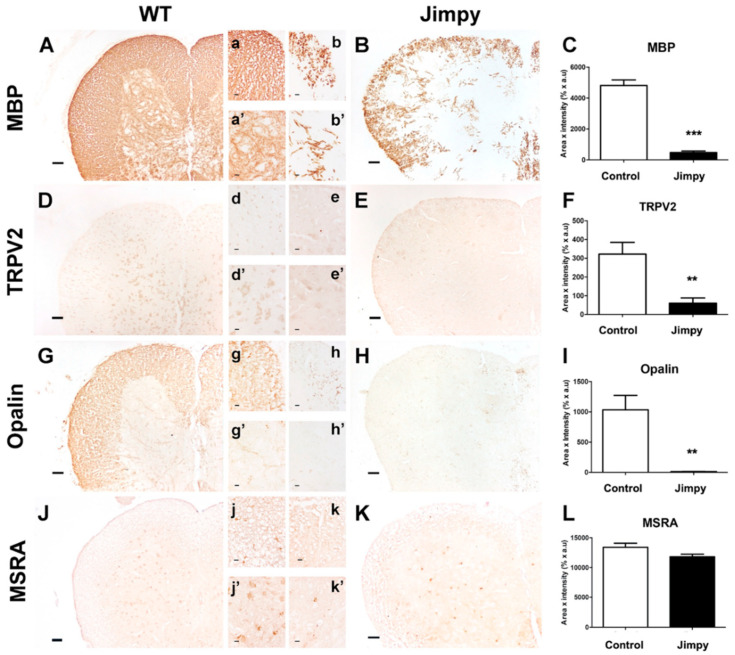
TRPV2, Opalin and MSRA expression in spinal cord of wild-type (WT) and hypomyelination jimpy mice. Immunohistochemical staining against MBP (**A**,**B**), TRPV2 (**D**,**E**), Opalin (**G**,**H**) and MSRA (**J**,**K**) were performed in spinal cord sections of 21-day-old WT and jimpy mice, and immunoreactivity was quantified and analyzed as Area x Intensity. Results show that MBP was importantly reduced in both GM (**a**,**b**) and WM (**a’**,**b’**) of jimpy mice compared to WT (**A**–**C**). TRPV2 expression was mainly located in neurons in GM (**d’**) and glial cells, putatively oligodendrocytes, and in WM (**d**) of WT. A significant decrease in TRPV2 expression in the spinal cord of jimpy mice was observed compared to WT (**E**,**F**,**e**,**e’**). Opalin expression was importantly found in WM of WT mice (**G**,**g**), and to a lesser extent also in GM (**g’**), but showed a marked decrease in WM and GM of jimpy mice (**H**–**I**,**h**,**h’**). MSRA was the only molecule that did not suffer an important reduction of its expression in both WM (**j**,**k**) and GM (**j’**,**k’**) in jimpy mice (**L**). Results were expressed as mean ± SEM. Statistical analysis of the results were performed with an unpaired Student’s *t*-test (** *p* < 0.01, *** *p* < 0.001). Scale bar (**A**–**K**) = 50 µm; (**a**–**k**,**a’**–**k’**) = 20 µm.

TRPV2, Opalin and MSRA protein expression was explored in spinal cord sections of control and jimpy mutant mice by immunohistochemistry. First, we determined the degree of hypomyelination in jimpy mice by staining the MBP, one of the most abundant proteins in myelin. As expected, MBP was highly expressed and homogenously distributed in the white matter (WM) of spinal cord sections of control mice (Figure 3A,a) and slightly expressed in grey matter (GM) (Figure 3a’). Jimpy mutant mice showed a clear reduction of MBP expression and myelin thickness in both WM and GM (Figure 3B,b,b’,C, *p* < 0.0001).

In basal conditions, TRPV2 expression in GM was mainly located in the cell body of spinal cord neurons, while in WM, it was in the soma of cells, putatively oligodendrocytes (Figure 3D,d,d’). In the hypomyelinating jimpy mutant mice, TRPV2 expression was significantly reduced in both neurons and oligodendrocytes (Figure 3E,e,e’,F, *p* = 0.0062).

We additionally evaluated Opalin expression in jimpy mice as an oligodendrocyte marker, but also as a TRPV2 interactor. Opalin is localized in WM-rich regions of the CNS and shows similar expression to the myelin marker MBP [13]. In the spinal cord of 21-day-old control mice we observed that Opalin was mainly distributed in WM, and located in cell bodies of oligodendrocytes and in the neuropil (Figure 3G,g). In GM, Opalin expression showed low expression, and was mainly located to some myelinated projections. As observed before for TRPV2 and MBP, Opalin was importantly reduced in both GM and WM (Figure 3H,h,h’) in the spinal cord of jimpy mice (*p* = 0.0092) in comparison with control animals (Figure 3I).

Since TRPV2 is activated through Met oxidation, which can easily be oxidized by reactive oxygen species (ROS) [20], we explored the expression of the Met reducing enzyme MSRA, a counteracting enzyme that partially reverses TRPV2 endogenous activation [2]. Immunostaining showed that MSRA was found in the GM and WM of both control and jimpy mutant mice (Figure 3J,K). In control mice, this enzyme was expressed mainly in glial cells, putatively oligodendrocytes, in WM, and in glial cells and neurons in GM. Similar observations were made in jimpy mutant mice (Figure 3j,k,j’,k’). Despite an apparent reduction of MSRA in jimpy mutant mice compared to control mice (Figure 3K), the quantitative analysis did not show significant differences between them (Figure 3L). 

### 2.5. TRPV2 and MSRA Expression Are Modulated in the Experimental Mouse Models of MS: The Cuprizone and the EAE Models

**Figure 4:**
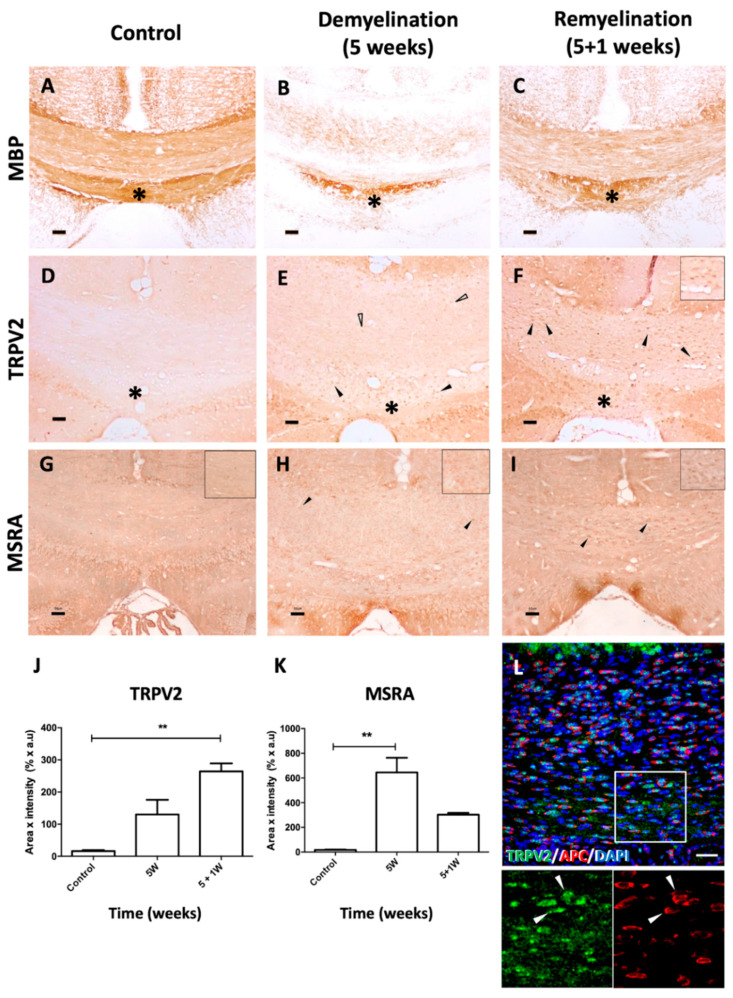
TRPV2 and MSRA expression in WM of cuprizone-induced demyelination and remyelination in mice. (**A**–**I**) Representative images of immunohistochemical staining directed against MBP, TRPV2 and MSRA in the CC of control mice (**A**,**D**,**G**) and cuprizone-intoxicated mice after demyelination (**B**,**E**,**H**, 5 weeks of treatment) and during remyelination (**C**,**F**,**I**, 5 weeks of treatment + 1 week of normal diet). (**A**–**C**) MBP immunostaining shows the myelinated CC of WT mice in basal conditions (**A**). At 5 weeks of cuprizone treatment, the CC is largely demyelinated (**B**) with a myelination-resistant area (*), and after one week of normal diet, the CC is mostly remyelinated (**C**). Control mice show undetectable levels of TRPV2 and MSRA in the CC (**D** and **G**). After demyelination, variably low and high levels of TRPV2 were observed in cell bodies (empty arrowheads and arrowheads, respectively in **E**,**F**). High levels of TRPV2 were clearly observed at remyelination (inset in **F**). During remyelination, most TRPV2+ cells co-expressed APC (full arrowheads in **L**), a marker for mature oligodendrocytes. In the CC area resistant to demyelination (* in **E**,**F**), TRPV2+ cells showed high levels of expression in both conditions. An increase in MSRA levels of expression were also observed in cell bodies after demyelination and remyelination (arrowheads in **H** and **I**). In demyelination, most cells expressed low levels of MSRA in the CC (inset in **H**), while during remyelination, a lower number of MSRA+ cells expressing higher levels of MSRA were observed (inset in **I**). (**J**–**K**) Quantification of TRPV2 and MSRA expression after cuprizone treatment. (**J**) Quantitative analysis of TRPV2 immunoreactivity in the CC confirmed the progressive increase of TRPV2 expression in demyelinating and remyelinating conditions compared to control mice, which was statistically significant in the latter. (**K**) Quantitative analysis of MSRA immunoreactivity in the CC showed that the increase of MSRA expression during demyelination was statistically significant when compared to control mice. Data are shown as ± SEM. Statistical analysis was performed by one-way ANOVA followed by Tukey’s multiple comparison test (** *p* < 0.01). Scale bar (**A**–**I**) = 50 μm; (**L**) = 100 μm.

To investigate TRPV2 and MSRA roles in demyelination, we used two experimental models of demyelination: the cuprizone-induced model and the EAE model. The effects of cuprizone intoxication on myelination were observed after MBP immunodetection: after a 5-week treatment, a substantial demyelination of specific WM areas, such as the corpus callosum (CC), was observed compared to basal conditions (Figure 4A,B), and cuprizone withdrawal allowed CC remyelination one week after (Figure 4C), as already quantified in previous work [22]. In the CC of control mice, TRPV2 and MSRA showed very low to undetectable levels (Figure 4D,G). After a 5-week cuprizone treatment, demyelinated CC showed a slight qualitative increase in TRPV2 (Figure 4E,H) that was mainly located in cell bodies of the CC, putatively oligodendrocytes (Figure 4E). In the caudal CC area, known to be resistant to myelination, TRPV2 increase was more visible (Figure 4D–F). After one week of remyelination, TRPV2 expression was significantly increased in mature oligodendrocytes of the CC when compared to control groups (*p* < 0.01) (Figure 4F, inset, J,K), as we confirmed with a double immunostaining against adenomatous polyposis coli (APC), and its levels were almost duplicated compared to demyelinated animals (Figure 4L). In the case of MSRA, a significant increase of this protein was observed after demyelination (*p* < 0.01) (Figure 4H,K), since most cells in the CC expressed low levels of MSRA. Later, after one week of remyelination, MSRA levels decreased, and only some MSRA+ cells were observed in the CC (Figure 4I,K).

**Figure 5:**
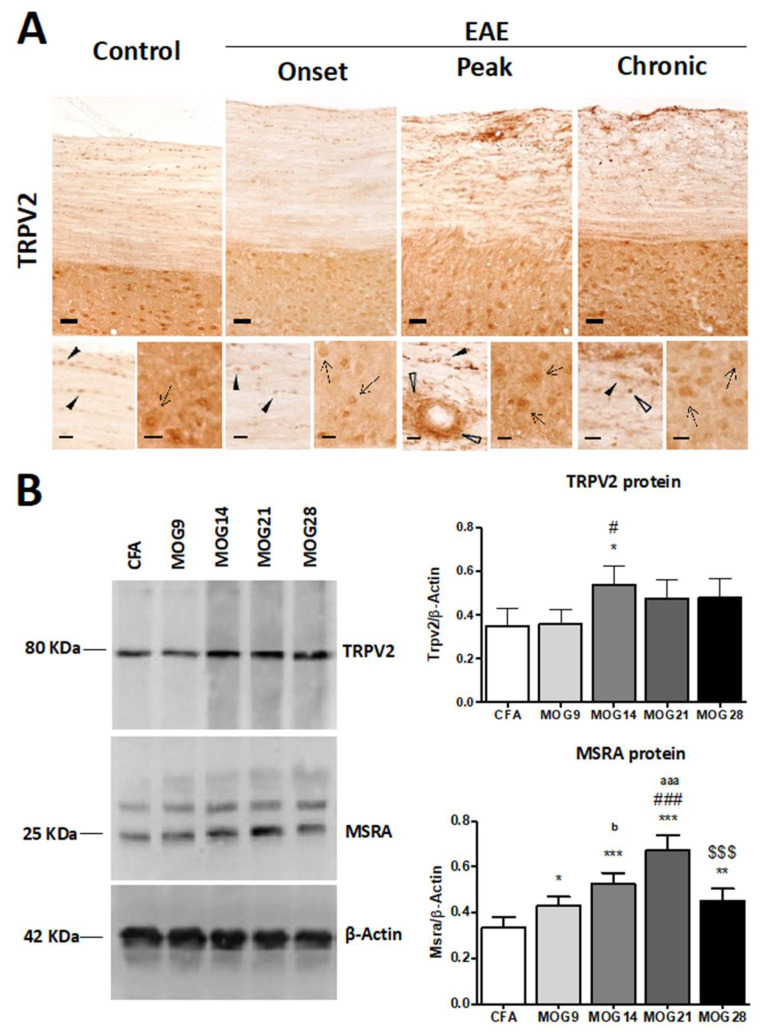
TRPV2 expression in spinal cord samples from WT and EAE mice. (**A**) Immunohistochemical staining of TRPV2 in spinal cord of control and EAE-induced mice in the onset, peak and in the chronic phase, during remission. In control conditions, TRPV2 is expressed in neurons in GM (inset, arrows), and in oligodendrocytes in WM (inset, arrowheads). After EAE induction, an increase in TRPV2 expression is mainly located in inflammatory focuses at the peak clinical symptomatology in WM (empty arrowheads). (**B**) MSRA and TRPV2 protein expression in thoracic spinal cord samples from CFA and MOG mice (9, 14, 21 and 28 days post-immunization). (**B**) A significant increase in TRPV2 protein levels (#, *p* = 0.0014) is determined in MOG14 mice when compared with CFA (*p* < 0.05) and when compared with MOG9 (*p* < 0.05). A significant increase (*p* < 0.05) in MSRA protein levels is observed in all groups of mice treated with MOG when compared with CFA mice, as well as between different groups treated with MOG: MOG9 vs. MOG21 (###, *p* < 0.001), MOG9 vs. MOG14 (^b^, *p* < 0.05), MOG21 vs. MOG28 (^$$$^, *p* < 0.001), MOG14 vs. MOG21 (^aaa^, *p* < 0.001). Bars show mean ± SEM; * *p* < 0.05, ** *p* < 0.01 and *** *p* < 0.001 vs. CFA using one-way ANOVA and Newman–Keuls post-test. Scale bars = 50 μm; (insets) = 20 μm.

In the EAE model, in which demyelination foci are developed in the spinal cord accompanied by clinical symptoms of paralysis, we analyzed TRPV2 expression in spinal cord sections. In control mice, TRPV2 was mainly expressed in neurons in GM and oligodendrocyte-like cells in the WM (Figure 5A). After EAE induction, in the onset of clinical symptomatology, similar patterns of TRPV2 expression were observed, but later, at the peak of clinical symptomatology, TRPV2 was mainly located in WM, in the inflammatory focuses and in infiltrated cells (Figure 5A). During the chronic phase of EAE, in the remission of clinical symptomatology, TRPV2 expression was still found in neurons in GM, and in oligodendrocyte-like cells and inflammatory focuses in WM. However, at this time point, an important loss of intensity was found in comparison to TRPV2 levels of expression observed in the peak (Figure 5A). Quantification of TRPV2 by western blot analysis (Figure 5B) confirmed the important increase of TRPV2 protein expression in the spinal cord of EAE mice at 14 days post-immunization (Figure 5B, *p* = 0.0014) compared to control mice. Later, at 21 and 28 days post-immunization, TRPV2 expression levels decreased and were maintained constant (Figure 5B). Evaluation of MSRA expression levels after EAE induction in mice showed an immediate significant increase when compared to the control complete Freund’s adjuvant (CFA)-injected mice (Figure 5B). Then, MSRA levels increased progressively until 21 days post-immunization (Figure 5B). At 28 days post-immunization, MSRA expression was significantly reduced when compared to 21 days post-immunization mice, and almost reverted to basal levels (CFA in Figure 5B).

### 2.6. MSRA and TRPV2 Gene and Protein Expression Are Modified in Frontal Cortex Samples of MS Patients

**Figure 6:**
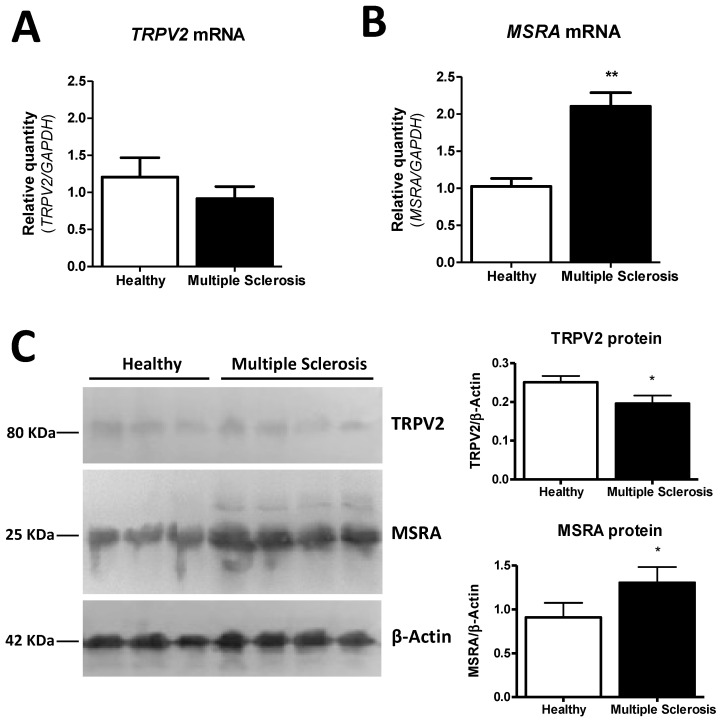
MSRA and TRPV2 expression in frontal cortex samples from healthy subjects (*n* = 4) and MS (*n* = 6) patients. (**A**) TRPV2 mRNA (*p* = 0.3314) is not changed between MS and healthy samples. (**B**) A significant upregulation in MSRA mRNA (*p* = 0.0014) is found in MS; (**C**) A significant decrease in TRPV2 protein levels (*p* = 0.0232) and a significant increase in MSRA protein levels (*p* = 0.0124) were determined in MS samples by western blot. Bars show mean ± SEM; * *p* < 0.05, ** *p* < 0.01 using unpaired Student’s *t*-test.

Gene and protein expression of TRPV2 and MSRA was explored in frontal cortex tissue samples of healthy (*n* = 4) and MS subjects (*n* = 6). In MS subjects, a non-significant tendency to decrease of TRPV2 mRNA levels was observed when compared to healthy subjects (Figure 6A). On the contrary, MSRA mRNA expression in MS subjects was found to be significantly upregulated (*p* = 0.0014) when compared to healthy subjects (Figure 6B). TRPV2 and MSRA protein levels were also determined by western blot (Figure 6C). Protein levels of both MSRA and TRPV2 showed the same tendency as the mRNA expression analysis (Figure 6A–C): while a significant decrease of TRPV2 protein levels was observed in MS patients when compared to the control (*p* = 0.0232) (Figure 6C), MSRA protein expression levels were also significantly increased in MS patients compared to healthy subjects (*p* = 0.0124) (Figure 6C).

## 3. Discussion

In this study we aimed to explore the role and expression of TRPV2 in the context of myelination disorders of the CNS, taking advantage of previous work indicating the potential role of TRPV2 and its interactors in myelination processes [11]. Opalin has been found in mature myelinating oligodendrocytes [23,24,25], but also at early stages of OPCs [26]. Opalin has not been found in microglia, astrocytes or neurons [23], and could be used as an oligodendrocyte lineage cell marker. Opalin and TRPV2 co-expression allowed for the identification of TRPV2+ oligodendrocyte-like cells. In physiological conditions, TRPV2 mRNA expression has been determined in OPCs, but not in oligodendrocytes [7]. Our results showed TRPV2 expression in mature oligodendrocytes of WM areas in the mouse spinal cord and brain in basal and inflammatory conditions. To the best of our knowledge, this is the first evidence describing TRPV2 expression in oligodendrocytes, together with the validation of the TRPV2–Opalin interaction pair. Although the physiological relevance of this interaction remains to be elucidated, the fact that TRPV2 is expressed in both microglia and oligodendrocytes opens the question of what the role of this channel is in microglia–oligodendrocyte cross-talk, and potentially in myelination.

The role of TRPV2 in microglia and phagocytosis under oxidative stress is slightly better understood so far. NO promotes TRPV2 translocation to the membrane which results in a higher microglia phagocytic capacity [5] and attenuated microglia proliferation [5]. In in vitro experiments, we have positively identified TRPV2+ microglia cells. In basal conditions, TRPV2 was found in the cell body of microglia and oligodendrocytes. Upon pro-inflammatory treatment with LPS, TRPV2 was translocated to the plasma membrane of both microglia and oligodendrocytes, and also colocalized with CD68+ intracellular compartments, in agreement with what has been previously shown in macrophages [27]. We measured the concentration of NO released in the cell media after all treatments; elevated levels of NO were especially found after LPS treatment. Indeed, TRPV2 is expected to be highly active after a pro-inflammatory stimulus, since several molecules produced by microglia, such as NO, or LPC-treatment and oxidative stress, may activate TRPV2 directly or indirectly [2,5,21]. Interestingly, we show that total TRPV2 levels decrease under pro-inflammatory conditions (LPS and LPC treatment) and increase under anti-inflammatory conditions (IL-4 treatment), where a decrease in NO concentration is found. Regardless of the mRNA levels derived from inflammatory conditions, TRPV2 protein was at the plasmatic cell membrane, and not in intracellular compartments, indicating that TRPV2 was ready to promote the TRPV2 NO-induced slow-onset calcium influx, leading to enhanced microglia phagocytic capacity, which is supported by previously published results [28].

NGF-dependent activation of endosomal TRPV2 in neurite outgrowth showed that TRPV2 can be active both at the plasma membrane and in intracellular compartments [7]. The neuronal TRPV2 role in axon and neurite outgrowth [8,9] has already been established, but there is no knowledge regarding TRPV2′s role in the oligodendrocyte–microglia cross-talk during myelination. Studies from Hainz et al. [14,15,16] using the pannexin-1 antagonist probenecid showed protective effects on MS murine models, such as preventing and arresting the progression of clinical symptoms in an EAE mouse model and diminishing demyelination in the cuprizone mouse model of demyelination/remyelination. Beyond being a pannexin-1 antagonist, probenecid is a known agonist for TRPV2 [1]. In addition, previous EAE results using tranilast, a well-known and specific TRPV2 antagonist [1], showed reduced mice paralysis when administered orally in an EAE mouse model [29]. In vitro experiments in this study, together with the bibliographic evidence above, bolster a strong argument to focus on the characterization of TRPV2 as a key player in myelination disorders. 

The jimpy mouse model, a pathophysiological model for PMD [17,18,19], shows a strong hypomyelination derived from X-linked deleterious mutations in the PLP gene. TRPV2 and PLP1 are putative interactors derived from our results in a yeast two-hybrid experiment [11], showing interaction at the biochemical level using purified TRPV2 (Figure 1A). Jimpy mice show severe hypomyelination, as we have seen in the current study with significantly decreased MBP expression (Figure 3A–C), failure in oligodendrocyte maturation, oligodendrocyte death, astrogliosis and microgliosis [30]. Observation of TRPV2 and Opalin expression in WT and jimpy mice reinforces a possible interaction of both proteins, since its expression was identified in the cell body of oligodendrocyte-like cells located in the WM of the spinal cord (Figure 3D–I). In jimpy mice, both TRPV2 and Opalin showed a significant reduction in WM. Taking into account the massive oligodendrocyte cell death observed in these animals [30], it is expected that an important reduction of both proteins would be found in hypomyelinating jimpy mice. Additionally in agreement with our previous results on cell cultures, the highly pro-inflammatory tissue environment found in hypomyelinating mice [31] may promote the reduction of TRPV2 levels, as observed in cell cultures (Figure 1 and Figure 2). In addition, other factors that could influence TRPV2 expression are the mutation in the PLP gene, a candidate that putatively interacts with TRPV2, and the oligodendrocyte maturation arrest, since, as we hypothesized based on previous evidence (and also supported by our results in the cuprizone model, discussed below), TRPV2 could be involved in myelination. At this point, it is worthwhile to highlight the choice of MSRA staining as an indirect measure of TRPV2 activity. As discussed earlier, endogenous activation of TRPV2 results from Met528 and Met607 oxidation in rat TRPV2 [2]. MSRA is in charge of reducing methionine-S-sulfoxide (Met-SO) to Met to protect cells from oxidative stress [32]. Thus, MSRA activity should be required to revert, at least partially, TRPV2 activation. In the jimpy mice, where TRPV2 is significantly reduced (Figure 3J–L), MSRA activity may not be affected due to the fact that the jimpy phenotype is not derived from an acute inflammatory process, as it happens in the cuprizone and EAE experimental models discussed below. 

In the experimental models of demyelination, the loss of myelin integrity was produced by oligodendrocyte cell death in the cuprizone model [33], or by an autoimmune reaction against CNS myelin proteins through the activation of autoreactive T cells in the EAE model [34,35]. Study of TRPV2 expression in the CC of mice with cuprizone-induced demyelination showed that TRPV2 progressively increased its expression, mainly in oligodendrocytes, in the peak of demyelination and in the first week of remyelination (Figure 4A–C). Since at those timepoints the main occurring event was the initiation of myelination of migrated OPCs [36], we could infer that TRPV2 is playing a role in the maturation of oligodendrocytes and myelination. Although a role for microglia should not be excluded, microgliosis and phagocytic activity reaches its peak of activity around 3–4 weeks [37], and taking into account that during remyelination of the CC, microglial activation and numbers decrease, a main role for TRPV2 in those microglial functions may not be expected at these timepoints. Interestingly, in remyelination, an anti-inflammatory micro-environment is found [38]. On the contrary, our results on TRPV2 expression in EAE support an important role for this ion channel in microglia and infiltrated immune cells, and as a result, in the pathology of this mouse model. TRPV2 was importantly upregulated in the inflammatory lesions, which are observed in the peak of symptomatology and are attenuated at the chronic phase [39]. Inflammatory lesions are mainly composed of microglia/macrophages, T cells, B cells and reactive astrocytes [40], all cells with previous reported expression on TRPV2 [5,6,10,41], and therefore, TRPV2 may be related to phagocytosis [5], proliferation or other unreported functions executed by those cells in the peak of EAE. In this acute phase, OPCs also proliferate as an attempt to react to demyelination, contrarily to mature oligodendrocytes that are reduced [42]. However, at the chronic phase, OPCs and mature oligodendrocytes are severely decreased, and only pre-myelinating oligodendrocytes remain stable [42]. Therefore, the stability in TRPV2 levels in chronic phases may be explained by the remaining pre-myelinating OPCs present in this chronic phase, since immunostainings also supported elevated TRPV2 expression in those cells. 

Indeed, the positive results in the reduction of demyelination in the cuprizone model and the amelioration of the EAE clinical symptoms after probenecid administration [14,15,16] may be explained by TRPV2 activation at several cellular levels: an increase of the phagocytic activity of microglia [5], which favors the elimination of cell debris and repair of the demyelinated area or other functions in infiltrating immune cells, and the enhancement of oligodendrocyte maturation and promotion of remyelination in TRPV2+ oligodendrocytes. Because of these multiple levels of activation in diverse cells, TRPV2 could be an interesting therapeutic target, as will be discussed later.

Evaluation of MSRA levels in our experimental models showed an expression pattern linked to inflammation and resolution. Oxidative stress is known to be elevated in all three models [31,43,44] and therefore the activation of MSRA is to be expected in those contexts. In the cuprizone and EAE models, MSRA was increased, coinciding with the peak of demyelination in the CC, and after the immune cell infiltration in the spinal cord, respectively. It should be taken into account that infiltrating and immune cells produce important amounts of free radical species that drive oxidative stress once they are activated. This increase in MSRA may be linked to a protection mechanism to reduce Met-SO to Met, since Met residues, which are easily oxidized by ROS [20], are capable of protecting cells from oxidative stress [45]. Furthermore, oligodendrocytes are the most sensitive CNS cells to oxidative stress [46,47] and MSRA may play an important counteracting and protective effect in these cells, as observed in its readily visible increase in oligodendrocytes in cuprizone-demyelinated mice. A different scenario was observed in hypomyelinating jimpy mice, as discussed earlier. Despite elevated levels of oxidative stress in mutant jimpy mice due to the inflammatory process, no modifications in MSRA levels were found in those animals. This could be the result of the important loss of cells observed in jimpy mice [30], which compensates for a possible increase on MSRA cell expression, and/or the inability of hypomyelinating mice to counterbalance oxidative stress effects by increasing MSRA.

To focus on TRPV2 and human myelin pathophysiology, we evaluated mRNA and protein expression of TRPV2 and MSRA in frontal cortex samples from MS patients compared to control subjects. In this case, a decrease in TRPV2 protein, which was non-significant in TRPV2 mRNA, was observed for MS patients compared to controls, while MSRA mRNA and protein expression were significantly increased in MS patients. Owing to the fact that ROS involvement has been described in MS pathogenesis [48,49], the increase in MSRA may respond to an attempt to protect cells from oxidative stress. These results support our previous findings in the experimental mouse models for MS: cuprizone and EAE. Unexpectedly, a downregulation of TRPV2 into the protein level was observed in the frontal cortex of MS patients compared to control subject samples. TRPV2 downregulation does not match with the upregulation of this protein observed in our results on the cuprizone and EAE models. However, it should also be considered that TRPV2 increase was observed in the acute inflammatory phase and during remyelination, while our MS patient samples should be contextualized in the chronic phase of this disease where inflammation is ameliorated and remyelination is absent. Therefore, this downregulation could be interpreted as the result of cell death observed in MS patients [50,51], or as the failure of oligodendrocytes to promote remyelination, thus approaching a scenario—with their respective important and characteristic pathological differences—similar to that observed in TRPV2 expression in the chronic inflammation of jimpy mice. Indeed, the downregulation of TRPV2 observed in MS patients suggests that TRPV2 may be playing a role, either as a cause or consequence, in this disease, and that promoting its specific activation/inhibition could be an interesting therapeutic target to be explored. Further studies on the expression of TRPV2 and its role in MS and other demyelinating diseases may unravel this question.

## 4. Materials and Methods

### 4.1. Animals

C57BL/6 males aged 8–10 weeks and females aged 2–4 months were used. Jimpy mutant male mice (jp/Y) were obtained and distinguished from normal control animals (+/Y) as described in Vela et al. [19]. Male jimpy mice aged 21 days and their corresponding male littermates (*n* = 6/group) were used. Animals were maintained with food and water ad libitum, in a 12 h light/dark cycle, at 22 ± 2 °C and 50–60% humidity. All experimental work was conducted according to Spanish regulations (Ley 32/2007, Real Decreto 1201/2005, Ley 9/2003, y Real Decreto 178/2004) in agreement with European Union directives (86/609/ CEE, 91/628/CEE i 92/65/CEE) and was approved by the Ethical Committee of the Autonomous University of Barcelona, Spain.

### 4.2. Cuprizone-Induced Demyelination and Remyelination in Mice

C57BL/6 males were fed with 0.2% (*w*/*w*) cuprizone (bis–cyclohexanone–oxaldihydrazone, Sigma-Aldrich, Missouri, MO, USA), as described in Petkovic et al. [22]. Three experimental groups were used: a control group fed with standard chow (*n* = 6), a demyelination group fed for 5 weeks with cuprizone (*n* = 7) and a remyelination group fed for one additional week with standard powdered chow (*n* = 6).

### 4.3. Experimental Autoimmune Encephalomyelitis in Mice

C57BL/6 female mice were immunized with a subcutaneous injection emulsion containing 100 μg/mouse of myelin oligodendrocyte glycoprotein (2) peptide 35-55 (MOG_35-55_, Espikem, Italy or M4939; Sigma-Aldrich, Missouri, MO, USA) and 1 mg/mouse of H37R M. tuberculosis (Difco, USA) in 200 μL of CFA (Sigma-Aldrich). Control sham-treated mice were injected with a similar emulsion without MOG35-55. All mice were injected intraperitoneally with toxin from *B. pertussis* (500 ng/mouse, Sigma-Aldrich) at 1 and 48 h after immunization. Clinical EAE symptoms were evaluated daily according to the following score: 0 = no symptoms; 0.5 = tail weakness; 1 = tail completely flaccid; 1.5 = mild difficulty in righting; 2 = great difficulty in righting; 2.5 = unsteady gait and paraparesis (mild paralysis of one or two hind limbs); 3 = complete paralysis of one hind limb; 3.5 = complete paralysis of one hind limb and mild paralysis of the other hind limb; 4 = paraplegia (complete paralysis of two hind limbs) and incontinence; 4.5 = paraplegia and mild paralysis of one or two forelimbs; and 5 = moribund or dead. CFA and MOG-EAE mice were killed at the following timepoints: 9 DPI (presymptomatic phase), 14 DPI (symptomatic phase), 21 DPI (EAE peak), 28 DPI and 40 DPI (chronic phase) and processed for immunohistochemistry (*n* = 2–3/group) or western blot (*n* = 4–5/group).

### 4.4. Human Samples

Human frontal cortex samples were obtained from the Tissue Bank at the Hospital Clinic (Barcelona, Spain). The whole procedure was performed in accordance with the Helsinki Declaration, the Convention of the Council of Europe on Human Rights and Biomedicine, and approved by the Ethical Committee of the University of Barcelona. Postmortem histological frontal cortex samples were obtained from control subjects and MS patients, as shown in Table 1.

### 4.5. FL and ARD TRPV2 Fragment Purification

Rat TRPV2 FL tagged with a C-terminal GFP and a 8xHis tag was overexpressed in yeast *Pichia pastoris* following our lab protocol as described in Suades et al. [52]. Yeast carrying the construct of interest was grown in YPD Zeocin at 30 °C. Cells were harvested and cultured in Minimal Glycerol Yeast media (MGY) for 48 h, till an Od600 of 10. Protein expression was induced by methanol, culturing the cells in Minimal Methanol media at 30 °C for 24 h. Cells were lysed using a bead beater. Lysates were centrifuged and pellet was solubilized for 1 h at 4 °C in 1% DDM, 0.2% cholesterol 50 mM Tris-HCl (pH 7.4) and 150 mM NaCl buffer complemented with EDTA-free protease inhibitor.

ARD of rat TRPV2 tagged with GFP and 8xHis tag in the C-terminus were heterologously expressed in *Escherichia coli* BL-21 strain, as described previously [53,54]. Expression of the protein was induced with 200 μM IPTG when the culture reached an OD600 of 0.6. After 5–6 h at 37 °C, cells were collected and lysed using a sonicator in 50 mM Tris-HCl (pH 7.4), 150 mM NaCl, 10% glycerol and EDTA-free protease inhibitors. 

All lysates were centrifuged for 45 min at 25,000× *g* and supernatant was collected and purified using Ni-NTA resin (30210, Qiagen, Hilden, Germany). See Supplementary Figure S4 for Rat TRPV2 FL.

### 4.6. Slot Blot 

FL rat TRPV2 and ARD TRPV2 domain were purified and placed at specific protein concentrations (see Figure 1 for details) into nitrocellulose membranes using a slot blot device. Membranes were dried for 30 min at room temperature (RT) and kept at 4 °C. For the interaction screen, TRPV2 membranes were blocked with blocking buffer (2% bovine serum albumin, BSA, in 0.1 M phosphate-buffered saline, PBS, pH 7.4) and incubated with a lysate from rat brain tissue at 1 μg/mL in blocking buffer for 1 h at RT. Then, membranes were washed in 0.1 M Tris-buffered saline (TBS, pH 7.4) + 0.1% Tween and analyzed by immunoblot against Opalin, Neurotrimin and proteolipid-1 proteins.

### 4.7. Cell Cultures

Secondary and quaternary cortical mixed glial cultures enriched in oligodendrocytes were obtained from 0- to 3-day-old WT C57BL/6 pups (*n* = 5), following the protocol described by Marignier et al. [55] with some modifications. Briefly, mice neocortices were dissected and digested with 0.25% trypsin (Gibco #25200-072) for 10 min at 37 °C. Trypsinization was stopped by adding an equal volume of culture medium composed of Dulbecco’s Modified Eagle Medium (DMEM) containing 4.5 g/l glucose (Gibco #41965-039), supplemented with 20% non-heat inactivated fetal bovine serum (FBS) (Gibco #10270106) and 0.1% penicillin/streptomycin with 0.02% deoxyribonuclease I (Sigma-Aldrich #D-5025). The solution was centrifuged at 200× *g* and the pellet obtained was resuspended in complete medium described above and brought to a single cell suspension by repeated pipetting followed by passage through a 100 μm pore mesh. Glial cells were seeded at a density of 2.0 × 10^5^ cells/mL in T25 cell culture flasks and were kept at 37 °C in humidified 5% CO_2_/95% air. After three days, a first medium replacement was performed with DMEM-High glucose-20% FBS. The medium was replaced every 5–6 days until the confluence of the cell culture was reached (after 14–16 days in vitro (DIV)). Once primary cultures were confluent, cells were washed for 5 min with 0.1 M PBS (pH 7.4—Gibco #14190094) and 2 mL of 0.125% trypsin-EDTA solution was added. After 5 min at 37 °C in humidified 5% CO_2_/95% air, trypsinization was stopped by adding 2 mL of complete medium. Cells were centrifuged for 7 min at 200× *g*, and the pellet was resuspended with a 1000 μL pipette for 3–5 steps. For secondary glial cultures, the cells were seeded at a density of 1.5 × 10^5^ cells/mL in T25 cell culture flasks and incubated at 37 °C in humidified 5% CO_2_/95% air. The medium was replaced every 5–6 days until cell confluence was reached (after 28–32 DIV). Quaternary cell cultures were obtained by repeating this process twice. For treatments, cells were seeded in 96- or 48-well culture plates (Falcon #353072, #353078) coated with poly-L-lysine (Sigma-Aldrich). 

### 4.8. Cell Culture Treatments

Cultures were treated for 24 h with 100 ng/mL of LPS from *E. Coli* (Sigma-Aldrich #L2654), 48 h with LPS+IFNγ (100 ng/mL LPS + 4 ng/mL IFNγ—Sigma-Aldrich #I4777), or 24 h with 20 ng/mL IL-4 (BioLegend #57430). LPC (4 ng/mL) was assayed for 24 h with quaternary cell cultures. Control cells were treated with an equivalent volume of culture medium. After treatment, conditioned media were removed and frozen. For immunocytochemistry, cells were fixed with 4% paraformaldehyde (PFA, Sigma-Aldrich #158127) in 0.1 M PBS for 20 min at RT. For ELISA, cells were removed and frozen.

### 4.9. Nitrite Assay

Nitric oxide (NO) production in the conditioned medium of cultures was assessed by the colorimetric Griess reaction. Twenty-five μL of conditioned medium from each culture and treatment were mixed with 50 μL of a 1% sulfanilamide solution (Sigma-Aldrich # S9251) and 5% phosphoric acid (PanReac Applichem #131032) for 5 min. Then, 50 µL of 0.1% N-(1-Naphthyl)ethylenediamine dihydrochloride solution (Sigma-Aldrich #222488) was added and incubated for 5 min at RT. Sodium nitrite (PanReac AppliChem #131703) was used to generate a standard curve (1.56–100 µM). Optical density at 530 nm was determined using a Varioskan™ LUX microplate reader (Thermo Scientific, Roskilde, Denmark). Triplicates were performed for each sample and treatment.

### 4.10. Immunocytochemistry

Cells were permeated with 25% methanol solution for 1 min, and washed afterwards with TBS. To block inspecificities, cells were treated for 15 min with TBS + 5% FBS (Biowest, Nuaillé, France, #S181H) and then incubated overnight at 4 °C with primary antibodies diluted in TBS + 5% FBS (Table 2). After several washes with PBS, cells were incubated for 1 h at RT with secondary antibodies diluted in TBS+5%FBS (Table 2) and nuclei counterstained with DAPI (1:10,000; Sigma-Aldrich #D9542). Microscopy images were obtained with an epifluorescence inverted microscope (Nikon ECLIPSE TE2000-E) at 20X with a digital camera connected to the microscope (Hamamatsu ORCA-ER monochrome microscope camera). Image collection was performed with MetaMorph Microscopy Automation and ImageJ Software (NIH, Bethesda, MD, USA). 

### 4.11. Colocalization Studies of Opalin and TRPV2 in Cell Cultures

At least four images were taken from cell cultures stained for TRPV2 (green) and Opalin (red) with and without LPS + IFNγ. Colocalization was evaluated using ImageJ and the JACoP plugin [56], which provided Pearson’s and Mander’s coefficient values (0 = no overlap; 1 = total overlap). The M1 (M_Opalin_) coefficient estimates the degree of red signal overlapping green, the M2 (M_TRPV2_) coefficient and vice versa.

### 4.12. Quantification of TRPV2 in Glial Cell Cultures by Enzyme-Linked Immunosorbent Assay

After washing with 0.1 M PBS, proteins were extracted using a RIPA buffer (1% NP40, 0.5% NA-deoxycholate, 0.1% SDS and one complete protease inhibitor cocktail tablet (Roche Diagnostics GmbH #11697498001) and quantified using BCA Assay (Pierce™ BCA Protein Assay Kit, Thermofisher #23227). Samples were stored at −20 °C until use. TRPV2 was quantified using the Mouse Transient Receptor Potential Cation Channel Subfamily V, Member 2 (TRPV2) ELISA Kit (ELISAGenie, #MODL01340) following the manufacturer’s protocol. Briefly, 100 μL of blank, standards (0.16 ng/mL–10.00 ng/mL) and samples (0.3 μg protein/μL) were added to wells and incubated for 2 h at 37 °C. After incubating the detection reagents and washing, the Substrate Solution was added for 15 min at 37 °C. Finally, 50 μL of Stop Solution was added and measurements were performed at 450 nm with an iMark^TM^ Microplate Reader (Bio-Rad, Munich, Germany), using Microplate Manager Software 6 (Bio-Rad).

### 4.13. Tissue Fixation and Processing for Immunohistochemistry

Following anesthesia of ketamine (80 mg/kg) and xylazine (20 mg/kg), animals were perfused intracardially for 10 min with 4% PFA in 0.1 M phosphate buffer (PB, pH 7.4). Brain samples of cuprizone-treated mice and spinal cords of jimpy mice were dissected and included in paraffin blocks, as previously described [22]. Ten-μm-thick coronal brain sections of cuprizone-treated mice and transversal spinal cord sections of jimpy mice were cut with a microtome. For EAE-induced mice and another group of cuprizone-treated mice, samples were prepared for cryostat sectioning. After spinal cord or brain dissection, samples were post-fixed with 4% PFA for 4 h at 4 °C, cryoprotected in 30% sucrose in 0.1 M PB for 48 h at 4 °C and frozen in ice-cold methylbutane (320404, Sigma-Aldrich). Thirty-μm-thick longitudinal spinal cord sections or coronal brain sections were obtained using a CM3050s Leica cryostat, and stored at −20 °C in antifreeze solution.

### 4.14. Immunohistochemistry

Brain and spinal cord paraffin sections and cryostat spinal cord sections were processed for immunohistochemistry. Paraffin sections were first deparaffined with xylol and hydrated with decreasing graded ethanol solutions, followed by an antigen retrieval step with sodium citrate buffer with 0.1% Triton (pH 6, 40 min at 95 °C). After washing with 0.1 M TBS, paraffin or cryostat sections were inactivated for endogenous peroxidase for either 5 or 10 min, respectively, with 2% H2O2 in a 70% methanol solution. Sections were washed with TBS+0.1% Triton (TBS-0.1%T) or TBS-1%T for cryostat sections, and incubated for 1 h with blocking buffer solution (BB; 0.05 M TBS, pH 7.4, containing 10% fetal calf serum, 3% BSA and 0.1% Triton X-100). Sections were incubated with the primary antibody diluted in BB overnight at 4 °C followed by 1 h at RT (Table 2). After washing, sections were incubated with the secondary antibody diluted in BB for 1 h at RT (Table 2). Then, sections were washed and incubated for 1 h at RT with horseradish peroxidase-conjugated streptavidin (1:500; SA-5004, Vector Laboratories, Burlingame, CA, USA), and reaction was visualized using a DAB Substrate Kit (SK4100, Vector Laboratories). Sections were dehydrated, treated with xylene and coverslipped with DPX. Sections incubated in BB without primary antibody were used as negative controls, and for MSRA staining, cerebellum sections were used as positive controls.

### 4.15. Quantification of Immunohistochemical Stainings

Quantitative analysis was performed on sections stained for MBP, TRPV2, Opalin and MSRA. Two to four sections per animal were analyzed. In spinal cord sections of jimpy mice, two photographs per section were taken at 10× magnification for MBP, Opalin and MSRA analysis, and at 20× magnification for TRPV2 analysis. In brain sections of cuprizone-treated mice (*n* = 3–4/experimental group), one photograph of the CC was taken at 10× magnification per section. All images were obtained using a DXM 1200F Nikon digital camera joined to a brightfield Nikon Eclipse 80i microscope and the corresponding ACT-1 2.20 software (Nikon Corporation, Tokyo, Japan). Quantification was performed using AnalySIS software (Soft Imaging System, Münster, Germany), manually setting the threshold for the staining. Analysis resulted in the percentage of area (% Area) and the intensity of the immunoreaction (Mean Gray Value Mean). AI index was calculated by multiplying the percentage of the immunolabeled area and the Mean Gray Value Mean [57]. Results of the AI index and intensity were expressed in arbitrary units.

### 4.16. Double Immunohistochemistry

Brain cryostat sections were processed for double immunohistochemistry for TRPV2 and APC (*n* = 3/group). After washes with 0.1 M TBS, sections were incubated for 1 h with BB, and then, overnight with rabbit anti-TRPV2 (1:100, Alomone) diluted in BB at 4 °C and 1 h at RT (Table 2). After washing with TBS-1%T, sections were incubated with an anti-rabbit secondary biotinylated antibody in BB for 1 h at RT (Table 2). Then, sections were washed and incubated for 1 h at RT with streptavidin conjugated to AF488 (1:500, S11223, Invitrogen). After washes, sections were blocked with BB for 1 h and incubated with the primary antibody mouse anti-APC (1:200) overnight at 4 °C and for 1 h at RT. After washes, an anti-mouse IgG secondary antibody conjugated to AF555 was incubated for 1 h at RT, and sections were counterstained with DAPI (1:10,000). Sections were mounted and coverslipped using Fluoromount G™ (0100-01, SouthernBiotech, Birmingham, AL, USA). Sections incubated in BB without primary antibody were used as negative controls. Stained sections were observed and photographed using a Zeiss LSM700 confocal microscope. Images were processed for the maximal intensity projection using Fiji software and merged using Adobe PhotoShop CS6 software.

### 4.17. Isolation of Total Proteins

Thoracic spinal cords from control (CFA) and EAE (MOG) mice were dissected and quickly frozen in dry ice. Tissues were first sonicated at 4 °C in RIPA buffer (PBS, 10 µL/mL; Igepal, 5 mg/m; sodium deoxycholate 1 mg/mL SDS) containing protease and phosphatase inhibitor cocktails (Sigma-Aldrich). One mL of ice-cold RIPA buffer was used per gram of tissue. After 30 min of incubation at 4 °C, samples were centrifuged at 5000× *g* rpm for 10 min at 4 °C and the supernatants were collected. For human samples, total protein extracts were obtained from the powder of frontal cortex samples using a mortar cooled with liquid nitrogen and 100 mg tissue homogenized in 1 mL of Laemmli buffer (0.125 M Tris-HCl pH 6.8, 2% SDS, 10% glycerol, 0.001% bromophenol blue and 5% 2-mercaptoethanol) [58,59] using a vortex. After that, samples were incubated for 10 min at 70 °C, centrifuged at 16,000× *g* for 10 min at RT and the supernatant was collected. 

Protein quantification was determined by Bradford assay (Bio-Rad Laboratories, Madrid, Spain). Samples were stored at −20 °C to be used for western blot analysis.

### 4.18. Quantification of TRPV2 and MSRA in EAE Mouse and Human Samples by Western Blot

Western blots were performed as previously described by Valente et al. [60]. Briefly, 30 µg of total protein was resolved on 10% SDS-PAGE gels (using the Bio-Rad Mini-PROTEAN 3 system) and transferred to a PVDF membrane (IPVH00010, Millipore, Darmstadt, Germany) for 90 min at 1 mA/cm^2^. Membranes were incubated overnight at 4 °C with primary antibodies (Table 2) diluted in immunoblot buffer (TBS containing 0.05% Tween-20 and 5% non-fat dry milk). After washing with immunoblot buffer, membranes were incubated for 1 h at RT with horseradish peroxidase-labeled secondary antibodies (Table 2). Membranes were developed with ECL-Plus (GE Healthcare, Little Chalfont, UK) and images were obtained using a VersaDoc System camera (Bio-Rad Laboratories). Data are expressed as the ratio between the band intensity of the protein of interest and the loading control protein (β-actin). 

### 4.19. RNA Extraction, cDNA Synthesis and Expression Analyses in Human Samples

Total RNA was extracted from 4 healthy subjects and 5 MS patients as previously described in [60]. Briefly, total RNA was isolated from frozen tissue samples using the Trizol method (Tri^®^ Reagent, Sigma-Aldrich). One microgram of RNA was reverse-transcribed with random primers using Transcriptor Reverse Transcriptase (Roche Diagnostics Scheiwz AG, Rotkreuz, Switzerland). Then, cDNA was diluted 1/10 to perform quantitative real-time polymerase chain reaction (qRTPCR) of MSRA, TRPV2 and GAPDH (housekeeping) mRNA with iTaq™ Universal SYBR^®^ Green Supermix (Bio-Rad #1725121) by qPCR in a Bio-Rad CFX384 RT-PCR System. Reaction mixtures totaling 15 μL contained 5 ng of cDNA, 7.5 μL of iTaq™ Universal SYBR^®^ Green Supermix, 500 nM of primer forward and 500 nM of primer reverse (Table 3). Conditions for a thermal cycling test consisted of one 3 min cycle at 95 °C, followed by 49 cycles of 10 s at 95 °C and 49 cycles of 30 s at 60 °C. Melting curves were completed at the end of the amplification with 1 cycle at 95 °C for 10 s, followed by a rate of increase of 0.5 °C/cycle from 65 to 95 °C. Primers were checked by melting curve analysis and no-template control reactions. The GAPDH gene was chosen as an internal reference. Expression levels were calculated using 2^−∆∆Ct^.

### 4.20. Statistical Analysis

Statistics was performed using Graph Pad Prism^®^ (Graph Pad Software Inc., San Diego, CA, USA) and results were expressed as mean ± Standard Error of the Mean (SEM). The statistical tests used were: for cell culture treatments, one-way ANOVA followed by Dunnett’s post hoc test (for LPS/IL-4 treatments) and paired Student’s *t*-test (for LPC treatment); for time-course experiments (cuprizone, EAE), one-way ANOVA with either Tukey’s or Newman–Keuls post hoc test, respectively; and for TRPV2–Opalin colocalization, jimpy mouse and MS human samples compared to controls, unpaired Student’s *t*-test.

## 5. Conclusions

**Figure 7:**
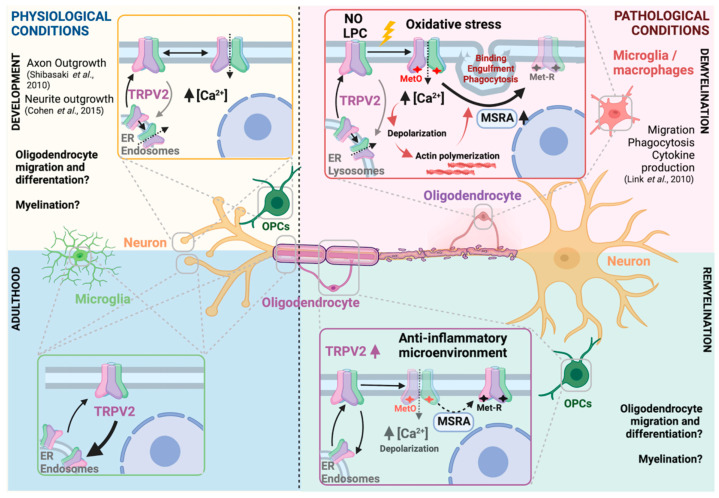
TRPV2-centered cellular cross-talk model in physiological and pathophysiological myelination. During embryonic development, TRPV2 develops an essential role in neurite and axon outgrowth. In that period, TRPV2 is known to be expressed and activated in neurons, and putatively in OPCs, where possible cross-talk between both cells might be triggered through TRPV2 activation. In adulthood, TRPV2 expression is found in microglia, neurons and oligodendrocytes. Upon an insult such as demyelination, TRPV2, as a non-selective ion channel, is activated. Some known triggering stimuli are NO, LPC or oxidative stress, the latter causing methionine oxidation which facilitates TRPV2 sensitization. TRPV2 activation promotes a sodium/calcium inward influx from extracellular media and/or ER or endosomes. Consequently, cells are depolarized, which initiate processes such as actin polymerization or phagocytosis in microglia or innate immune cells. In those cells, TRPV2 has been involved in migration, phagocytosis or cytokine production. TRPV2 inactivation is promoted by MSRA enzyme, by reducing oxidized methionine. In our results, we observed both an increase of TRPV2 and MSRA in demyelinating scenarios. During remyelination, OPCs proliferate and migrate to the demyelinated area, where they differentiate through several stages to mature oligodendrocytes, and start myelination. Our results showed increased TRPV2 expression during this period. Overall, ubiquitous TRPV2 expression and activation in de- and remyelinating processes suggest a multicellular cross-talk that promotes myelin repair at all levels. TRPV2 is relevant in development and demyelination, making this model suitable for the recapitulation hypotheses. Created with BioRender.com (accessed on 10 January 2022) [8,9,10].

Previous studies have shown that TRPV2 is expressed mainly in neurons at developmental stages (Figure 7). In this work, we first identified TRPV2 expression in oligodendrocytes, and its regulation during pro-inflammatory and anti-inflammatory conditions in vitro. This regulation was also observed in deleterious myelination disorders in mice, such as hypomyelination and de-/remyelination, suggesting the involvement of TRPV2 in the cross-talk between neurons, microglia and oligodendrocytes. TRPV2 regulation was predominantly found during inflammation in microglia and immune cells, and during remyelination in oligodendrocytes (Figure 7). Finally, we also identified TRPV2 as an altered protein in the pathology of MS, which points out TRPV2 as an interesting clinical target in myelination disorders’ therapy. Further studies are needed to better understand the role of TRPV2 in myelination diseases and how TRPV2 modulation could affect the phenotype of these diseases.

## Figures and Tables

**Table 1:** Post-mortem human samples of frontal cortex obtained from healthy and MS patients.

Human Sample	Gender	Age (Years Old)	Post-Mortem Time (h)	Multiple Sclerosis Type	Neurological/Histopathological Evaluation
Control #1	Male	66	7	-	Absence of histological lesions; Absence of neurological disease
Control #2	Male	70	n.d.	-	Absence of histological lesions; Absence of neurological disease
Control #3	Female	74	3.33	-	Absence of histological lesions; Absence of neurological disease
Control # 4	Female	81	23.5	-	Few Aβ-plaques in entorhinal area; Absence of neurological disease
Multiple Sclerosis #1	Female	65	8	Secondary progressive	Some chronic active lesions; Many chronic inactive lesions
Multiple Sclerosis #2	Female	48	8.25	Secondary progressive	Few chronic active lesions; Many chronic inactive lesions
Multiple Sclerosis #3	Male	46	3.25	Primary progressive	Many chronic active lesions; Few chronic inactive lesions
Multiple Sclerosis #4	Male	68	7	Secondary progressive	Few chronic active lesions; Many chronic inactive lesions
Multiple Sclerosis #5	Female	52	3.5	Secondary progressive	Very few chronic active lesions; Many chronic inactive lesions

**Table 2:** List of antibodies and reagents used in the slot blot, immunocytochemistry (ICC), immunohistochemistry (IHC), double immunofluorescence (IF) and western blot (WB).

Primary Antibody	Host	Dilution	Reference Manufacturer	Secondary Antibody	Dilution	Reference, Manufacturer
*Slot blot*						
Opalin	Goat	1:1000	sc-163187, SantaCruz Biotechnology	Donkey anti-goat IgG-HRP	1:2000	sc-2033, SantaCruz Biotechnology
NTM	Rabbit	1:1000	sc-98979, SantaCruz Biotechnology	Goat anti-rabbit IgG-HRP	1:2000	sc-2030, SantaCruz Biotechnology
PLP	Rabbit	1:1000	sc-98781, SantaCruz Biotechnology	Goat anti-rabbit IgG-HRP	1:2000	sc-2030, SantaCruz Biotechnology
*ICC*						
GFAP	Mouse	1:1000	G3893, Sigma-Aldrich	Alexa 555 donkey anti-mouse	1:1000	A31570, Invitrogen
CD68	Rat	1:250	MCA1957, AbD Serotec	Alexa 555 goat anti-rat	1:1000	A21434, Invitrogen
PDGFRα	Rat	1:100	558774, BD Biosciences	Alexa 555 goat anti-rat	1:1000	A21434, Invitrogen
TRPV2	Rabbit	1:200	sc-31155, SantaCruz Biotechnology	Alexa 488 donkey anti-rabbit	1:1000	A21206, Invitrogen
Opalin	Goat	1:50	sc-163187, SantaCruz Biotechnology	Biotinylated horse anti-goat	1:500	BA-9500, Vector Laboratories
*IHC*						
TRPV2	Rabbit	1:200	ACC-032, Alomone	Biotinylated horse anti-rabbit	1:500	BA-1100, Vector Laboratories
MBP	Rabbit	1:200	A-623, Dako	Biotinylated horse anti-rabbit	1:500	BA-1100, Vector Laboratories
MSRA	Rabbit	1:200	ab16803, abcam	Biotinylated horse anti-rabbit	1:500	BA-1100, Vector Laboratories
Opalin	Goat	1:50	sc-163187, SantaCruz Biotechnology	Biotinylated horse anti-goat	1:500	BA-9500, Vector Laboratories
*Double IF*						
TRPV2	Rabbit	1:100	ACC-032, Alomone	Biotinylated horse anti-rabbit	1:500	BA-1100, Vector Laboratories
APC	Mouse	1:200	Calbiochem (#OP80)	Alexa 555 donkey anti-mouse	1:500	A31570, Invitrogen
*WB*						
TRPV2	Goat	1:500	PA5-18989, ThermoFisher	Mouse anti-goat IgG-HRP	1:2000	AP186P, Sigma-Aldrich
MSRA	Rabbit	1:1000	ab16803, abcam	Donkey anti-rabbit IgG-HRP	1:5000	A16035, ThermoFisher
β-actin	Mouse	1:100,000	A1978, Sigma-Aldrich	Goat anti-mouse IgG-HRP	1:2000	62-6520, ThermoFisher

**Table 3:** Primers used for quantitative polymerase chain reaction (qPCR).

Target Gene	Primer Sequences (5′→3′)	Annealing Temp. (°C)
MSRA	F: GGC CAT CTA CCC GAC CTC T R: GCC ATT GGG GTT CTT GCT CA	60
TRPV2	F: TCA GGT TGG AGA CAT TAG ATG GA R: TCG GTA GTT GAG GTT GAC TCT T	60
GAPDH	F: CAT GAG AAG TAT GAC AAC AGC CT R: AGT CCT TCC ACG ATA CCA AAG T	60

## Data Availability

Not applicable.

## Supplementary Materials

The following supporting information can be downloaded at: https://www.mdpi.com/article/10.3390/ijms23073617/s1.
