# Supplementary material for: TRPV2: A Key Player in Myelination Disorders of the Central Nervous System
Source: Int J Mol Sci. 2022 Mar 25;23(7):3617. doi: 10.3390/ijms23073617 (PMC8999035; doi:10.3390/ijms23073617)
Supplement: Supplementary file 1 [file ijms-23-03617-s001.zip › ijms-1644413-supplementary.pdf]

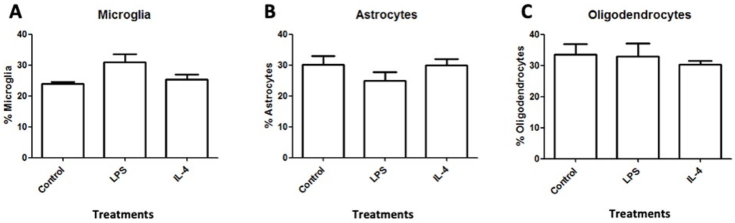

Supplementary Figure S1.Determination of glial populations in mixed glial secondary cell cultures in basal conditions and after pro-inflammatory LPS and anti-inflammatory IL-4 treatments.

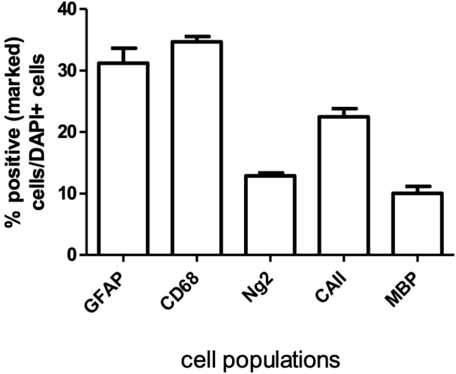

Supplementary Figure S2. . Characterization of mixed glial cultures under basal conditions.

Western Blot Quantification	Healthy (H)			MEAN	Multiple Sclerosis (MS)				MEAN	Unpaired t test (H vs MS)
	H1	H2	H3		MS1	MS2	MS3	MS4		
MSRA	0,878	0,873	1,016		1,277	1,344	1,246	1,290		
TRPV2	0,280	0,263	0,227		0,238	0,221	0,176	0,152		
Correction Factor (β-Actin)	1,027	1,063	0,946		1,067	1,026	1,008	0,863		
MSRA β-Actin-Normalized	0,856	0,821	1,074	0,917±0,137	1,196	1,309	1,236	1,495	1,309±0,133	*, p = 0,0124
TRPV2 β-Actin-Normalized	0,273	0,248	0,240	0,253±0,017	0,223	0,216	0,175	0,176	0,197±0,026	*, p = 0,0232

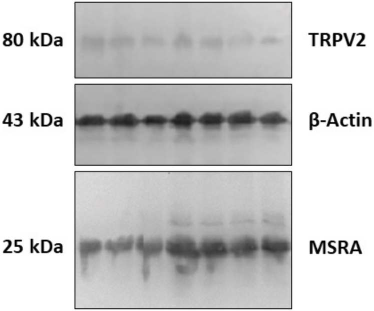

Supplementary Figure S3. Protein determination and data analysis of immunoblot in Figure 6C.

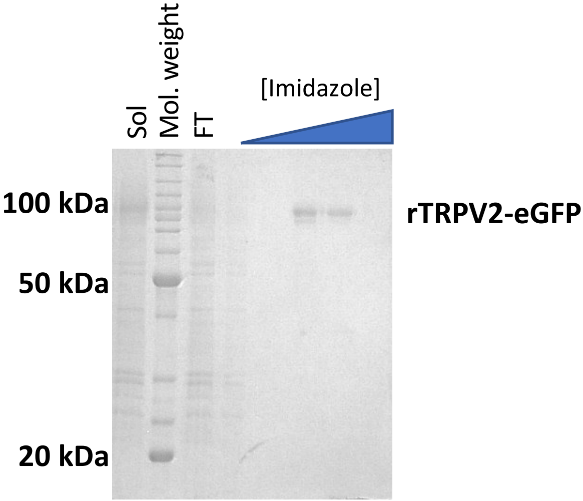

Supplementary Figure S4. Coomassie blue staining of Ni-NTA FL-TRPV2 enrichment and imidazole elution resolved in a 12% SDS-PAGE electrophoresis. Sol; detergent solubilized fraction loaded in the chromatography column. FT; Flow through fraction.
